# Adult Presentation of Dyke–Davidoff–Masson Syndrome, a Radiological Enigma: A Case Report

**DOI:** 10.1155/crra/5550152

**Published:** 2025-04-03

**Authors:** Suman Paudel, Ankit Acharya, Rhijuta Pokharel, Prerana Singh Rokaha, Pratik Singh Rokaha, Ashaya Luitel

**Affiliations:** ^1^Department of Radiology, Kathmandu Medical College Public Limited, Kathmandu, Nepal; ^2^Kathmandu Medical College Public Limited, Kathmandu, Nepal; ^3^Lumbini Medical College and Teaching Hospital, Tansen, Nepal

## Abstract

**Introduction and Importance:** Dyke–Davidoff–Masson syndrome (DDMS) is a rare neurological condition characterized by focal or generalized drug-resistant epilepsy, hemiparesis, face or body asymmetry with atrophy, and cognitive impairment in early childhood and adulthood. DDMS is generally diagnosed in the paediatric age group. Neuroimaging shows skull bone thickening with cerebral hemiatrophy and hyperpneumatization of sinuses.

**Case Presentation:** Here is a case of a middle-aged female presenting with a history of multiple episodes of seizure since childhood. MRI showed diffuse atrophy of the left cerebral hemisphere with hypertrophy of the contralateral hemisphere, hyperpneumatization of the left frontal sinus, and thickened calvaria, all characteristics of DDMS. Based on the history, clinical findings, and MRI reports, it was diagnosed as a case of DDMS.

**Discussion:** DDMS can be due to injury to the brain, either intrauterine or during early childhood. The features can be confused with other conditions like Rasmussen encephalitis, hemiconvulsion-hemiplegia-epilepsy (HHE syndrome), Sturge–Weber syndrome, Silver–Russell syndrome, basal ganglia germinoma, Fishman syndrome, and linear nevus syndrome. Before making a diagnosis, a proper antenatal and postnatal history with early childhood presentations should be taken. Occupational therapy, physiotherapy, and seizure control improve the patient's quality of life.

**Conclusion:** Though DDMS is usually diagnosed during early childhood, a few missed cases lead to later findings in life, resulting in late medical consults and affecting an individual's lifestyle. Management includes only symptomatic relief. Paediatricians, radiologists, neurologists, and gynaecologists need to be well-informed about the case for its early diagnosis and management.

## 1. Introduction

Dyke–Davidoff–Masson syndrome (DDMS) is a rare neurological condition that was first described in 1933 by Dyke, Davidoff, and Masson [1]. DDMS is a paediatric condition that predominantly affects males [2, 3]. Only a few cases of adult DDMS have been reported to date [4]. Clinical manifestations include focal or generalized drug-resistant epilepsy, hemiparesis, face or body asymmetry with atrophy, and cognitive impairment, which are typically present in individuals in their early childhood and adult years [5]. Neuroimaging techniques such as magnetic resonance imaging (MRI) and computed tomography (CT) scans are used to diagnose the condition and help exclude differential diagnosis [5]. Neuroimaging characteristics of DDMS include hyperpneumatization of the paranasal sinuses, thickening of the skull bone, and cerebral hemiatrophy [5]. The exact mechanism of DDMS is not entirely elucidated; however, DDMS causes are attributed to be both congenital and acquired, potentially leading to brain injury in the early developmental phase during intrauterine life or early years of childhood [6, 7]. Here, we present a case of adult DDMS in a middle-aged female.

## 2. Case Presentation

A middle aged, unmarried, nonsmoker, nonalcoholic, south Asian female presented to our emergency room with a history of multiple episodes of abnormal body movements associated with uprolling of the eyeball, tongue bite, and frothing from the mouth. Birth history as well as family history was unremarkable.

Her medical history included a febrile seizure at the age of 1 year. A diagnosis of meningitis was made at that time, and the patient was treated with antibiotics. She was discharged after 10 days of inpatient stay. She did, however, continue to experience seizures following this event, and she was prescribed antiseizure medicine (oral carbamazepine) for 2 years.

After this, she was seizure-free for several years. However, 5 years before this presentation, the patient had generalized tonic–clonic seizure for which she was again prescribed with oral carbamazepine for 1 month. After this, the patient was seizure-free for 5 years until the current presentation.

On neurological examination, the Glasgow Coma Scale (GCS) was 15/15, sensory examination was intact, and motor examination revealed muscle strength of 4/5 (Modified Research Council [MRC] grade) on the right upper and lower limb. The right upper and lower limb muscles had mild atrophy with exaggerated tendon reflexes. Also, right-sided facial palsy (upper motor type) was present. All the lab reports including serum electrolytes were within the normal limits. EEG was performed, but no active seizure focal was found. No genetic analysis was performed because of patient refusal. An MRI brain was done for further evaluation, the findings of which are described below.

**Figure 1:**
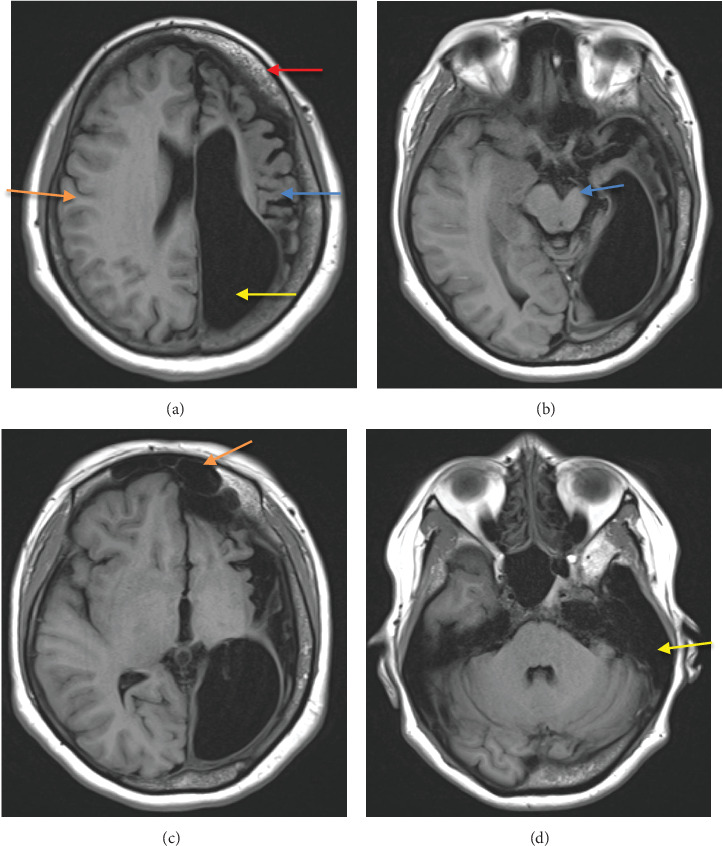
The T1-weighted axial image at the level of the central semiovale shows diffuse left cerebral atrophy (blue arrow [a]) with hypertrophy of the right cerebral hemisphere (orange arrow [a]), ex vacuo dilatation of the left lateral ventricle (yellow arrow [a]), and diffuse left calvarial thickening (red arrow [a]). The T1-weighted axial image at the level of the midbrain shows atrophy of the left cerebral peduncle consistent with Wallerian degeneration (blue arrow [b]). The T1-weighted axial image at the level of the frontal sinus shows hypertrophy of the left frontal sinus (orange arrow [c]). The T1-weighted axial image at the level of the bilateral petrous apex shows hypertrophy of the left mastoid sinus (yellow arrow in d).

**Figure 2:**
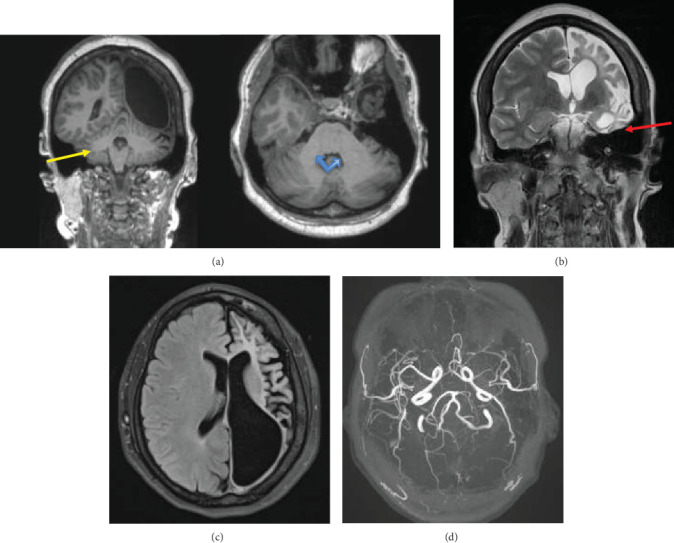
T1-weighted coronal and axial images show atrophy of the right cerebellar hemisphere compared to the left (yellow arrow [a]) with a smaller right middle cerebellar peduncle compared to the left (blue arrow [a]). The coronal T2-weighted image at the level of the petrous apex shows elevation of the left petrous ridge (red arrow [b]). The T2 FLAIR axial image shows no evidence of abnormal signal intensity in the atrophic left cerebral hemisphere (c). The noncontrast 3D-TOF MRA MIP image shows normal cerebral vessels (d).

**Figure 3:**
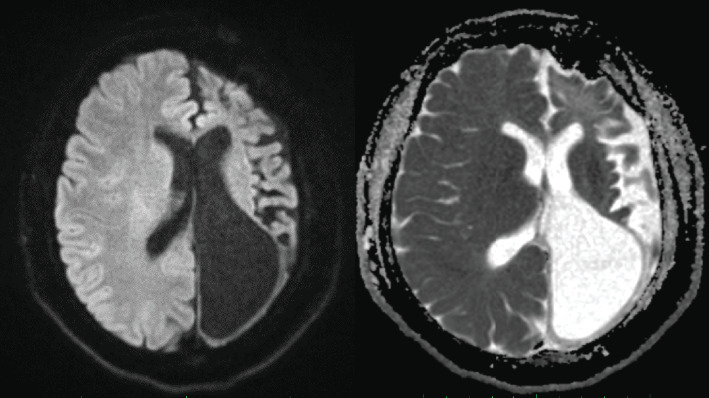
Axial DWI and corresponding ADC map images show no evidence of diffusion restriction within the atrophic left cerebral hemisphere.

An MRI brain with noncontrast three-dimensional time-of-flight (3D-TOF) magnetic resonance angiography (MRA) was performed using the standard institutional protocol. It showed diffuse atrophy of the left cerebral hemisphere including atrophy of deep gray matter (basal ganglia and thalamus) with hypertrophy of the contralateral cerebral hemisphere and ex vacuo dilatation of the left lateral ventricle (Figure 1a). Widening of the sulcal space of the left cerebral hemisphere along with dilatation of left lateral ventricle was seen. Diffuse calvarial thickening was seen on the left side compared to the right (Figure 1a). Atrophy of the left side of the brainstem including the left cerebral peduncle and left side of pons was seen representing Wallerian degeneration (Figure 1b). Hypertrophy of the left frontal sinus and left mastoid air cells was present (Figure 1c,d). Diffuse atrophy of the right cerebellar hemisphere was seen representing crossed cerebellar diaschisis (Figure 2a). Elevation of the left petrous ridge was seen compared to the right (Figure 2b). There was no evidence of abnormal signal intensity on T2 and fluid-attenuated inversion recovery (FLAIR) images (Figure 2c). Also, no evidence of restricted diffusion was seen on diffusion-weighted imaging (DWI) (Figure 3). MRA showed normal cerebral vessels (Figure 2d).

All the clinical findings, neuroimaging findings, and history contributed to diagnosing DDMS.

The patient was managed symptomatically with intravascular paracetamol and intravascular levetiracetam. Her vitals were regularly monitored and was discharged with oral levetiracetam 500 mg twice daily and follow-up advice.

The patient has been advised for a regular follow-up in the neuromedicine outpatient department.

## 3. Discussion

DDMS is an uncommon diagnosis; only 21 adult cases out of over 100 cases have been documented to date [4]. First reported by Dyke, Davidoff, and Masson in 1933, the radiographic signs of pneumatic encephalographic anomalies of the skull were linked to clinical symptoms of refractory seizures, facial asymmetry, hemiparesis, and intellectual disability in a case series of nine patients [1]. Other symptoms observed in earlier research included hemiplegia, psychological issues, learning and speech difficulties, and cognitive impairment [8, 9]. Studies have shown that DDMS is more common among males and paediatric age groups, but our case is of a female adult in her 5th decade [2, 3]. The clinical and radiological features of our case are consistent with DDMS. Numerous case reports show both congenital and acquired etiopathogenesis, such as vascular malformation, infection, and trauma during the prenatal and early childhood brain development period [6, 7, 10]. This patient has a normal birth history but a history of bacterial meningitis at the age of 1 year, following which she developed refractory seizures and other features of decreased cognitive ability.

The severity of the initial injury determines the clinical characteristics that patients with DDMS display [4]. Neuropsychiatric and psychiatric symptoms have also been reported in a case study done by Sordia-Ramirez et al. in 2019, which was associated with systemic lupus erythematosus [11]. So, a detailed history including past, antenatal, postnatal, and paediatric history is required for the diagnosis of the syndrome. A physical and mental assessment should also be thorough to rule out any other similar illness. In our case, the patient showed generalized tonic–clonic seizure, with right-sided weakness, but with normal neuropsychiatric and psychiatric findings.

It is crucial to rule out other similar disorders such as Rasmussen encephalitis, hemiconvulsion-hemiplegia-epilepsy (HHE syndrome), Sturge–Weber syndrome, Silver–Russell syndrome, basal ganglia germinoma, Fishman syndrome, and linear nevus syndrome before making a diagnosis of DDMS [5, 12, 13].

Rasmussen encephalitis is characterized by severe, intractable focal epilepsy and cognitive impairments that usually manifest in childhood. A distinct imaging feature includes unilateral hemisphere atrophy without noticeable calvarial changes in the skull (without thickened calvaria, and hyperpneumatization of sinuses), unlike our case of DDMS [10, 12–14].

Three stages comprise the presentation of HHE syndrome: focal clonic seizures, hemiplegia, and epilepsy [15]. During infancy or early childhood, the symptoms steadily worsen, starting with unilateral convulsive seizures and progressing to temporary or permanent hemiplegia and epilepsy [16]. The features provided in HHE are also very similar to DDMS, so it is necessary to differentiate the two conditions.

The hallmark of Sturge–Weber syndrome is cerebral atrophy associated with cortical calcifications and leptomeningeal angiomas [13, 17]. Seizures, cognitive impairment, and weakness on one side of the body are common symptoms [17]. Facial birthmark that resembles a port-wine stain, tram-track calcification in the brain, absence of midline shift, and absence of calvarial thickening are all important signs of Sturge–Weber syndrome, none of which were present in this case [17, 18].

Mental retardation, seizures, hemiparesis, and skin lesions on the scalp, face, or neck are typical symptoms of the uncommon, spontaneous neurocutaneous illness known as linear nevus sebaceous syndrome [19]. Left hemimegalencephaly (HME) and unilateral ventricular dilatation that resembles cerebral hemiatrophy are usually associated with it [7, 10, 19]. The dermatological findings help to differentiate the condition from DDMS.

Silver–Russell syndrome is characterized by growth retardation, delayed maturation of the bones, clinodactyly, normal cranial size, average cognitive ability, unusual facial morphology (including a triangle-shaped face, large forehead, small pointed chin, and slim wide mouth), and hemihypertrophy of cerebral hemisphere [7].

Unilateral cranial lipoma and an eye lipodermoid are the hallmarks of Fishman syndrome, often referred to as encephalocraniocutaneous lipomatosis, a rare neurocutaneous condition. Neuroimaging frequently shows a calcified cortical area and hemiatrophy, and it usually manifests as seizures [14].

Cerebral hemiatrophy with progressive hemiparesis is also a common feature of basal ganglia germinoma, a rare brain tumor. However, imaging shows calvarial abnormalities, isolated hemorrhages, modest surrounding edema, and cystic regions [14].

The management of DDMS aims to treat symptoms through targeted interventions. Individualized occupational therapy, physical therapy, and speech therapy are provided to patients. With a success rate of 85% in certain situations, hemispherectomy is the preferred treatment for kids with hemiplegia and intractable disabling seizures [5].

## 4. Conclusion

Although DDMS is commonly diagnosed in paediatric group patients, few cases have also been described in adult patients. The lack of expertise in the related field can delay the diagnosis. Therefore, paediatricians, neurologists, radiologists, and gynaecologists should be well aware of the condition for an early diagnosis and management. The management options include occupational therapy, physiotherapy, and seizure control. An early recognition of the syndrome can greatly improve the outcome and lifestyle of the patient.

## Data Availability

The authors have nothing to report.
