# Supplementary figures and images for: Adult Presentation of Dyke–Davidoff–Masson Syndrome, a Radiological Enigma: A Case Report
Source: Case Rep Radiol. 2025 Apr 3;2025:5550152. doi: 10.1155/crra/5550152 (PMC11991835; doi:10.1155/crra/5550152)

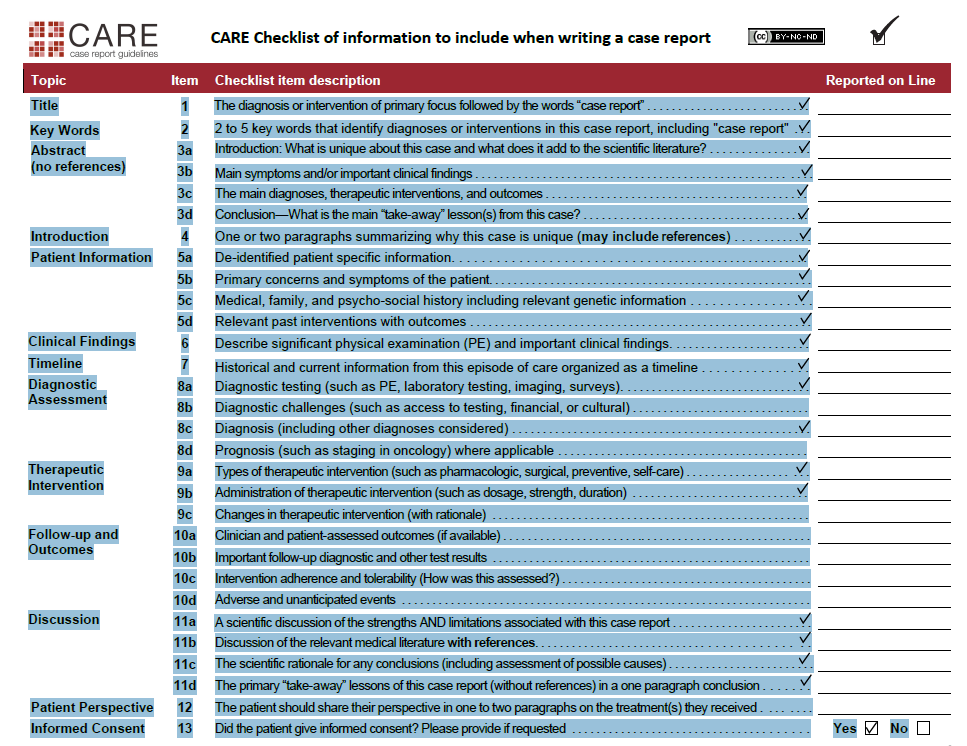

Supplement: Supporting Information — Additional supporting information can be found online in the Supporting Information section. Care checklist. [file 5550152.f1.docx]
